# Identification of potential biomarkers of gold nanoparticle toxicity in rat brains

**DOI:** 10.1186/1742-2094-9-123

**Published:** 2012-06-12

**Authors:** Nikhat J Siddiqi, Mohamed Anwar K Abdelhalim, Afaf K El-Ansary, Abdullah S Alhomida, W Y Ong

**Affiliations:** 1 Department of Biochemistry, College of Science, King Saud University, P.O. Box 22452, Riyadh, 11495, Saudi Arabia; 2 Physics & Astronomy, College of Science, King Saud University, P.O. Box 2455, Riyadh, 11451, Saudi Arabia; 3 Department of Anatomy, National University of Singapore, Singapore, 119260, Singapore; 4 Neurobiology and Ageing Research Program, National University of Singapore, Singapore, 119260, Singapore

**Keywords:** Gold nanoparticles, Oxidative stress, Antioxidant enzyme, DNA damage, Cell death, Interferon-γ, Neurotransmitters

## Abstract

**Background:**

Gold nanoparticles (AuNPs) are finding increased use in therapeutics and imaging. However, their toxic effects still remain to be elucidated. Therefore this study was undertaken to study the biochemical effects of AuNPs on rat brain and identify potential biomarkers of AuNP toxicity.

**Methods:**

Male Wister rats weighing 150–200 g were injected with 20 μg/kg body weight of 20-nm gold nanoparticles for 3 days through the intraperitoneal route. The rats were killed by carbon dioxide asphyxiation 24 h after the last dose of gold nanoparticle injection. The parameters studied included lipid peroxidation, glutathione peroxidase, 8- hydroxydeoxyguanosine, caspase-3, heat shock protein70, serotonin, dopamine, gamma amino-butyric acid and interferon-γ.

**Results:**

In this study AuNPs caused generation of oxidative stress and a decrease of antioxidant enzyme, viz., glutathione peroxidase activity in rat brain. This was accompanied by an increase in 8-hydroxydeoxyguanosine, caspase-3 and heat shock protein70, which might lead to DNA damage and cell death. Gold nanoparticles also caused a significant decrease in the levels of neurotransmitters like dopamine and serotonin, indicating a possible change in the behavior of the treated animals. There was a significant increase in the cerebral levels of IFN-γ in treated animals.

**Conclusion:**

This study concludes that AuNPs cause generation of oxidative stress and an impairment of the antioxidant enzyme glutathione peroxidase in rat brain. AuNPs also cause generation of 8-hydroxydeoxyguanosine (8OHdG), caspase-3 and heat shock protein70 (Hsp70), and IFN-γ, which may lead to inflammation and DNA damage/cell death.

## Background

The emerging field of nanobiotechnology offers the potential for the development of exquisitely sensitive diagnostics and organ/tumor-targeted therapies [1]. Over the past few decades, there has been considerable interest in developing nanoparticles as effective drug delivery devices [1]. Gold nanoparticles (AuNPs) are expected to have a wide range of applications in the future because they are easy to synthesize and have good biocompatibility. Studies of Sonavan et al. 2008 [1] have demonstrated a wide distribution of gold particles inside the living system. Their studies have shown that 15–50-nm AuNPs can cross the blood–brain barrier, resulting in their accumulation in the brain [1]. The biosafety of metallic gold in well known, and it has been in *in vivo* use since the 1950s [2]. However, it is essential to understand the interaction of gold nanoparticles with vital organs such as the brain, liver, kidneys, heart, etc. The toxicity of nanoparticles *in vivo* is determined by many parameters, such as dose, routes of exposure, metabolism, excretion, and immune response. The toxicological profiles of nanomaterials are determined by their chemical composition, size, shape, aggregation and surface coating [2]. Nanoparticle technology also holds promises to treat diseases like Alzheimer’s and Parkinson’s diseases. Nevertheless, effective targeting of drugs to the brain remains a challenge because of the restrictive properties of the blood–brain barrier (BBB). This barrier, predominantly formed by endothelial cells that are physically joined by tight junctions in their external membranes, limits the molecular exchange to transcellular transport, thus restricting the passage of molecules across the barrier. The healthy BBB also largely protects the brain from blood-borne nanoparticle exposure; however, a number of pathologies, including hypertension and allergic encephalomyelitis, have been shown to increase BBB permeability to nanoparticles [3]. The widespread use of nanoparticles in the future is likely to have an enormous impact on human health. Therefore, it is essential to understand the effects of nanoparticles on the brain, which is one of the vital organs of the body. This study was undertaken to investigate the effect of AuNPs on rat brain and identify potential biomarkers of AuNP toxicity.

## Methods

### Animals

Male Wister rats weighing 150–200 g were obtained from the animal house of the Pharmacy College of King Saud University, Riyadh. After 1 week of acclimatization, the rats were injected with 20 μg/kg body weight of 20-nm gold nanoparticles for 3 days through the intraperitoneal route. The rats were killed by carbon dioxide asphyxiation 24 h after the last dose of gold nanoparticle injection. Ethical animal care guidelines were followed.

### Chemicals

All the chemicals used were purchased from Sigma Chemical C., St Louis, MO, USA. Double-distilled water was used throughout the study.

### Gold nanoparticles (AuNPs)

Gold nanoparticles of 20 nm (Product MKN-Au-020, Canada) in aqueous solution of 0.01% gold concentration were used in this study.

### Preparation of sample

The rats were killed by carbon dioxide asphyxiation 24 h after the last dose of gold nanoparticle injection. The brains were dissected out, washed in ice-cold saline and homogenized in saline (10% weight/volume) at 4^0^ C. The homogenates were centrifuged at 3,000 rpm for 10 min in a cooling centrifuge. The supernatant was used in the study.

### Parameters related to oxidative stress

#### *Lipid peroxidation*

Lipid peroxidation was measured by measuring the level of malondialdehyde (MDA) by the method of Buege and Aust, 1978 [4]. 0.25 ml of homogenate was mixed with 0.75 ml of double-distilled water and incubated in a shaking water bath at 37^0^ C for 1 h. The reaction was stopped by adding 1.5 ml of 20% trichloroacetic acid. The reaction mixture was centrifuged at 3,000 rpm for 10 minutes. Then 1 ml of 0.67% thiobarbituric acid was added to 1 ml of the supernatant, and the reaction mixture was incubated at 100°C for 10 min. On cooling absorbance was read at 535 using a reagent blank.

Values were expressed as moles of thiobarbituric-acid reactive substances formed/h/mg protein.

### Glutathione peroxidase (GPx)

GPx activity was determined by the method of Wendel A, 1981 [5], with modifications. The mixture contained 0.6 ml of 0.25 M phosphate buffer (pH 7.0), 0.3 ml of 10 mM GSH, 0.3 ml of 10 mM EDTA, 0.3 ml of 10 mM sodium azide, 0.3 ml of 2 mM NADPH and 20 μl glutathione reductase. The mixture was added to 0.9 ml of the sample and incubated at 30 °C for 5 min. The reaction was initiated by the addition of 0.3 ml of 2.5 mM hydrogen peroxide. The absorbance was immediately read at 340 nm.

### Parameters related to DNA damage and apoptosis

#### *8- hydroxydeoxyguanosine (8OHdG)*

8-Hydroxydesoxyguanosine was determined using the Bluegene Elisa kit from Life Sciences Advanced Technologies, Inc., Saint Petersburg, FL, USA, using the manufacturer’s instructions.

### Caspase-3

Caspase-3 was measured using an ELISA kit from Cusabio. The concentration of caspase-3 was determined according to the manufacturer’s instructions.

### Heat shock protein70 (Hsp70)

Hsp70 was measured in homogenates of brain using an ELISA kit (Uscn Life Science Inc., Wuhan, China) according to the manufacturer’s instructions.

### Neurotransmitters

#### *Assay of serotonin*

Serotonin was measured using an ELISA kit, a product of Immuno-Biological Laboratories, Inc. (IBL-America), Minneapolis, MN, USA.

### Dopamine

Dopamine was extracted by using a cis-diol-specific affinity gel, acylated and then derivatized enzymatically. Quantitative assay was performed using an ELISA kit, a product of Immuno-Biological Laboratories, Inc. (IBL-America).

### Assay of gamma amino-butyric acid (GABA)

Quantitative determination of GABA was done using an ELISA immunoassay kit, a product of ALPCO, UK.

### Parameter related to inflammation

#### *Interferon-γ (IFN-γ)*

IFN-*γ* was measured using an ELISA kit, a product of Thermo Scientific, Waltham, MA, USA, according to the manufacturer’s instructions.

### Statistical analysis

A computer SPSS program was used, and the results were expressed as mean ± SD (*n* = 6). Comparisons were made by the one-way ANOVA between the control and treated groups. Dunnett’s test was used to compare between the groups. Receiver operating characteristic analysis was done. Area under the curve, specificity and sensitivity were calculated.

## Results and discussion

Neurons are nerve cells that, together with neuroglial cells, constitute the nervous tissue making up the nervous system. A neuron consists of a nerve cell body (or soma), axon and dendrites. Neurons receive nerve signals (action potentials), integrate action potentials and transmit the signals to other neurons [6]. Although the human nervous system is much more specialized and complicated than that of lower animals, the structure and function of neurons is essentially the same in all animals [6]. *In vitro* systems to study the effects of nanoparticles on the nervous system have included neuron and nanoparticle cultures to determine the effects on neuronal functions [7]. Endpoints could include reactive oxygen species/reactive nitrogen species production, apoptosis, metabolic status, effects on the action potential and ion regulation in general [7]. Human neural cells, such as hippocampal cells in the central nervous system, are the most sensitive and delicate cells in bioorganisms, and are responsible for brain functions and emotions. They are vulnerable to ischemia, oxygen deficiency and external factors. One of the concerns in science and technological development in the twenty-first century is that nanoparticles may produce potential functional and toxic effects on human neural cells owing to their ability to pass through biological membranes [8].

The high proportion of surface atoms/molecules can give rise to a greater chemical as well as biological activity, for example the induction of reactive oxygen species in cell-free medium as well as in cells [9]. Nanoparticles are able to translocate across cell barriers from the portal of entry (e.g., the respiratory tract) to secondary organs, and to enter cells by various mechanisms and associate with subcellular structures. This makes nanoparticles uniquely suitable for therapeutic and diagnostic uses, but it also leaves target organs such as the central nervous system vulnerable to potential adverse effects (e.g., oxidative stress) [9]. In general, translocation rates of NPs from the portal of entry into the blood compartment or the central nervous system (CNS) are very low. Important modifiers of translocation are the physicochemical characteristics of NPs, most notably their size and surface properties, particularly surface chemistry [9]. Increased surface reactivity would imply that the nanoparticles would exhibit increased biological activity. The increased biological activity of nanoparticles could be useful to penetrate cells for drug delivery. However, undesirable effects of nanoparticles could include generation of oxidative stress and/or impairment of antioxidant defense responses.

**Figure 1:**
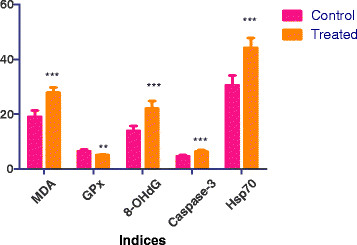
**Effect of gold nanoparticles on lipid peroxidation, glutathione peroxidase, 8- hydroxydeoxyguanosine, caspase-3 and heat shock protein 70 in rat brain.** ***P < 0.001 Dunnett’s test.

In the present study gold nanoparticles caused significant generation of oxidative stress in the brain. This is evident by an increased lipid peroxidation of 46% (*P* < 0.001) in the brains of rats treated with gold nanoparticles when compared to the brains of control rats (Figure 1). Malondialdehyde (MDA) is the end product of lipid peroxidation, and an increase in the level of MDA suggests higher lipid peroxidation. Oxidative stress has been identified as a likely mechanism of nanoparticle toxicity [10].

**Figure 2:**
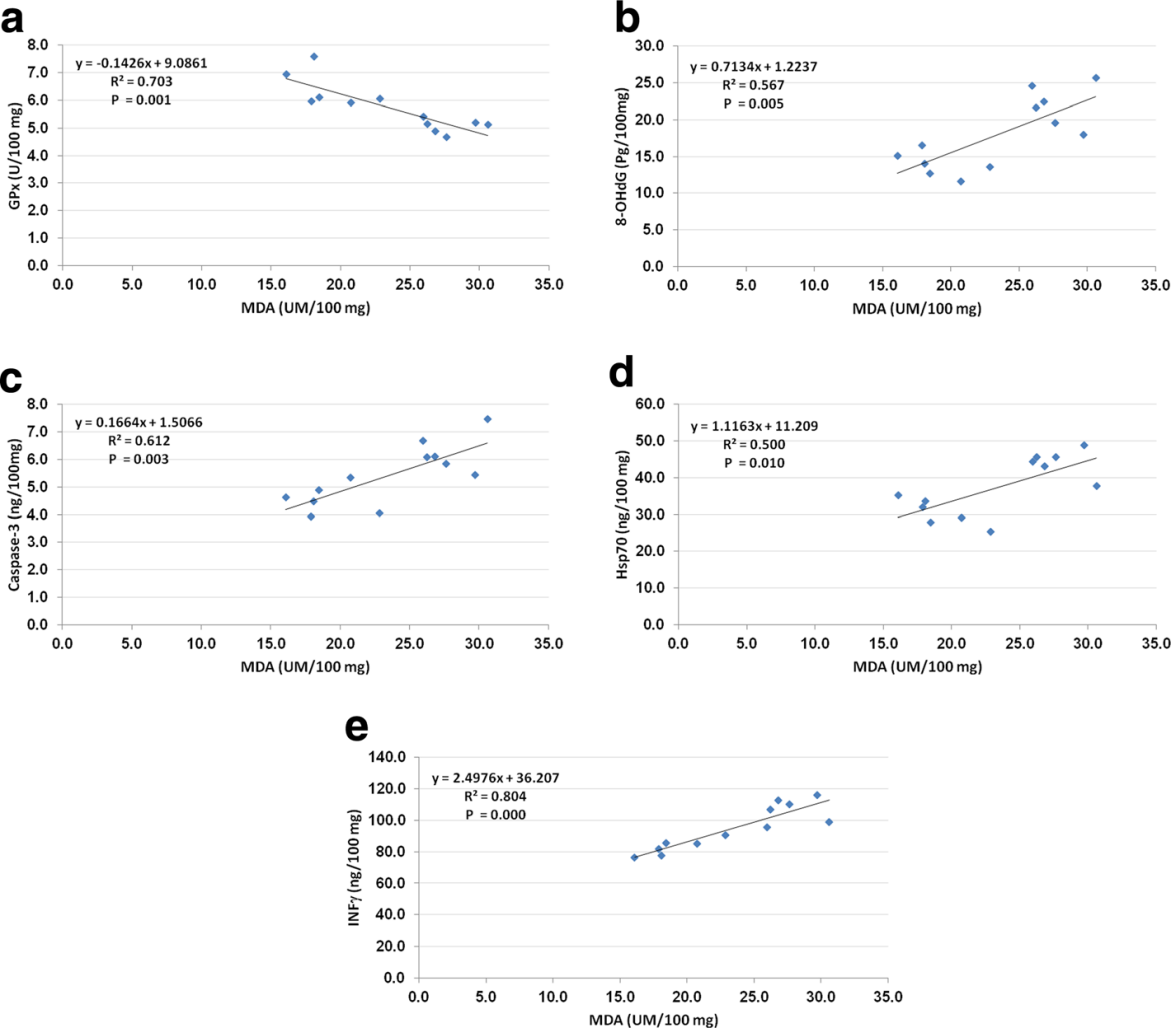
**(a) Correlation between MDA (UM/100 mg) and GPx (U/100 mg) with best fit line curve (negative correlation).** (**b**) Correlation between MDA (UM/100 mg) and 8-OHdG (Pg/100 mg) with best-fit line curve (positive correlation). (**c**) Correlation between MDA (UM/100 mg) and caspase-3 (ng/100 mg) with best-fit line curve (positive correlation). (**d**) Correlation between MDA (UM/100 mg) and Hsp70 (ng/100 mg) with best-fit line curve (positive correlation). (**e**) Correlation between MDA (UM/100 mg) and INFγ (ng/100 mg) with best-fit line curve (positive correlation).

Increased malondialdehyde production was accompanied by a significant decrease (*P* <0.001) of 21% glutathione peroxidase in the brains of treated rats when compared to control group of rats (Figure 1). Glutathione peroxidase (GPx) is one of the important antioxidant enzymes involved in protecting the cell against oxidative stress. The glutathione redox cycle is a major source of protection against mild oxidative stress [11]. GPx, a selenium-containing antioxidative enzyme scavenging system, acts directly as an antioxidant and an inhibitor of lipid peroxidation. GPx has been reported to protect the cell against peroxidative damage [12]. In this study there was a negative correlation between MDA and GPx, which might indicate an impairment of antioxidant defenses in the brain by the free radicals generated by AuNP (Figure 2a).

Gold nanoparticles also caused a significant increase in 8-hydroxydeoxyguanosine (8OHdG), caspase-3 and heat shock protein 70 (Hsp70) by 57%, 38% and 45%, respectively, in the brains of treated rats when compared to the control group (Figure 1). There was also a positive correlation between MDA and 8-hydroxydeoxyguanosine (8OHdG), caspase-3 and heat shock protein 70 (Hsp70) (Figures 2b, c, d). Oxidative stress is considered one of the foremost reasons for DNA damage. Reactive oxygen species generated in metabolizing cells could attack DNA base guanine forming the 8-OHdG lesions, which is known to have mutagenic potential and hence used routinely as a biomarker for carcinogenesis [13]. AuNPs have been reported to induce DNA damage in the form of single-strand lesions in human lung fibroblasts [14].

The brain is particularly susceptible to oxidative stress-induced damage because of its (1) high oxygen consumption to maintain metabolism, (2) high content of polyunsaturated fatty acids with their oxidatively susceptible allylic hydrogen atoms, (3) relatively high concentration of iron and ascorbate to carry out the radical-generating Fenton reaction, and (4) relatively low concentration of antioxidants and antioxidant enzymes [15].

Caspases are crucial mediators of programmed cell death (apoptosis) [16]. Among them, caspase-3 is a frequently activated death protease, catalyzing the specific cleavage of many key cellular proteins. Pathways to caspase-3 activation have been identified that are either dependent on or independent of mitochondrial cytochrome c release and caspase-9 function. Caspase-3 is essential for normal brain development, and is important or essential in other apoptotic scenarios in a remarkable tissue-, cell type- or death stimulus-specific manner. Caspase-3 is also required for some typical hallmarks of apoptosis, and is indispensable for apoptotic chromatin condensation and DNA fragmentation in all cell types examined [16]. In the present study, a significant increase in caspase-3 along with 8OHdG (Figure 1) may indicate activation of apoptotic machinery in the brain after nanoparticle injection. Zehendner et al. (2011) have also implicated caspase-3 in various neurodegenerative disorders [17].

Heat shock proteins play a role in protecting cells from damage generated by a variety of stress. Different cytoprotective effects attributed to heat shock proteins include refolded and misfolded proteins, degradation of unstable proteins, prevention of protein aggregation, etc. [18]. In the present study AuNPs caused a significant increase in Hsp70 (Figure 1), which may be due to the generation of oxidative stress in the brain. There was a positive correlation between the malonaldehyde generated as a result of lipid peroxidation and Hsp70 (Figure 2d).

**Figure 3:**
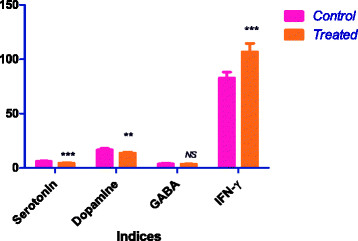
**Effect of gold nanoparticles on serotonin, dopamine, gamma aminobutyric acid and interferon-γ in rat brain.** ***P < 0.001 Dunnett’s test.

Dopamine and serotonin are neurotransmitters, which are found in neurons of all animals. Alteration in the normal expression of these transmitters is associated with human neurological disorders such as Parkinson’s disease and depression [19,20]. AuNPs also cause a significant decrease in serotonin (*P* < 0.001) and dopamine (*P* <0.01), but no significant change in gamma amino butyric acid (Figure 3) in rat brains. Serotonin has been reported to have an inhibitory action in brain [21]. Dopamine is a neurotransmitter involved in modulating aggressive behavior in animals [22]. AuNPs have been shown to activate neurotransmitter enzymes like 1-cholineesterase and monoamine oxidase [23].

**Figure 4:**
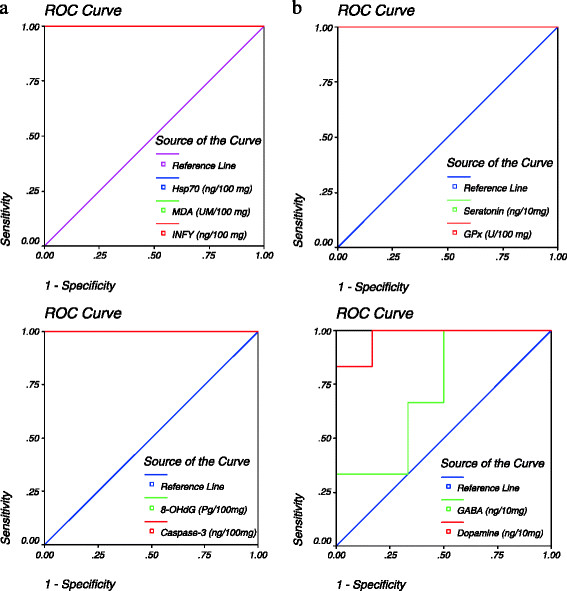
**(a) ROC curve of INFγ (ng/100 mg), MDA (UM/100 mg), Hsp70 (ng/100 mg), caspase-3 (ng/100 mg) and 8-OHdG (Pg/100 mg) in gold nanoparticle-treated group.** (**b**) ROC Curve of GPx (U/100 mg), serotonin (ng/10 mg), dopamine (ng/10 mg) and GABA (ng/10 mg) in gold nanoparticle-treated group.

Gold nanoparticle injection also caused a significant increase (*P* < 0.001) in the level of interferon γ when compared to the control group of rats (Figure 3). The increase in the concentration of interferon γ demonstrates the inflammatory potential of AuNPs [24]. Palladium nanoparticles have been shown to induce IFN-γ release and increase the expression of IFN- γ mRNA from peripheral blood mononuclear cells [25]. Figure 4 shows ROC analysis of the measured parameters. The results demonstrate that IFN-γ, MDA, HSP70, caspase-3 and 8-OHdG demonstrated more than 80% sensitivity and therefore can be used as biomarkers of gold nanoparticle toxicity.

**Figure 5:**
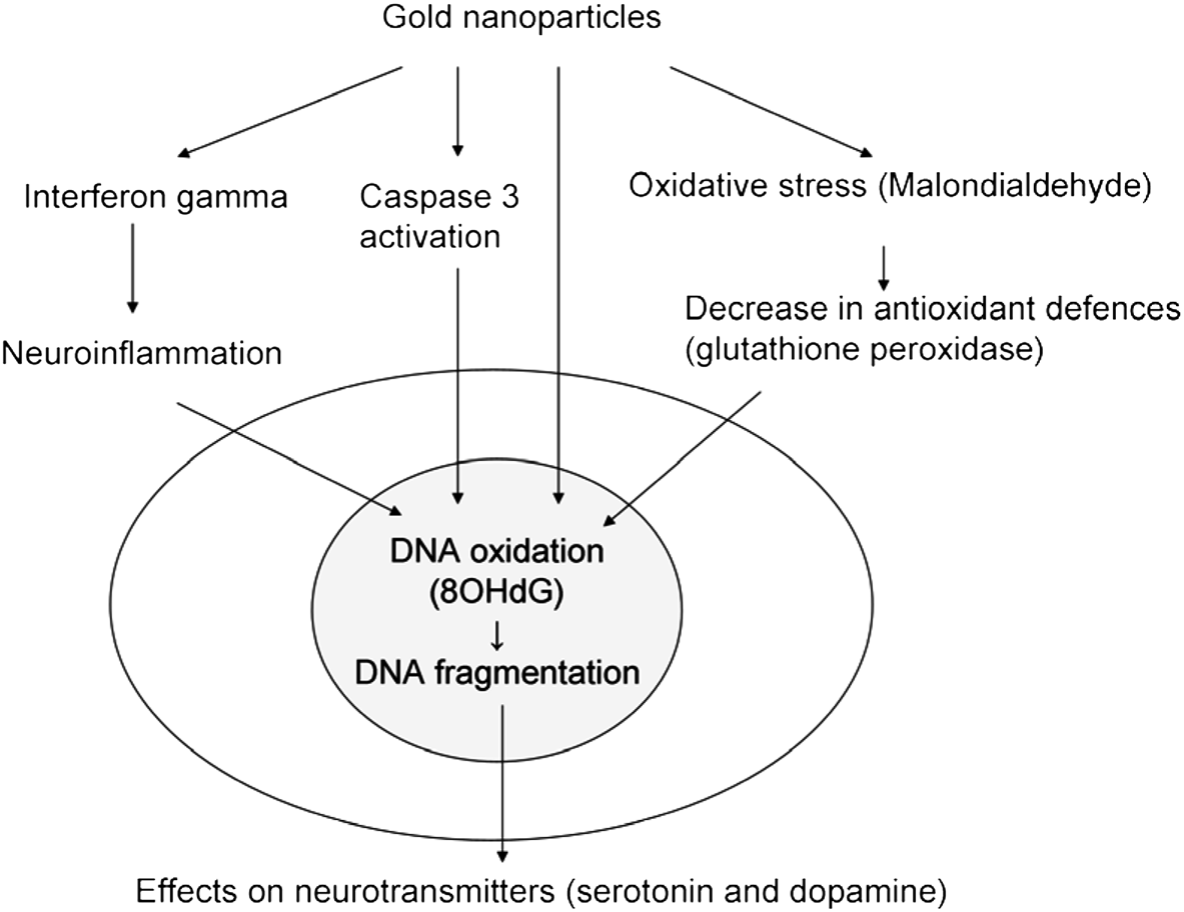
Effects of gold nanoparticles on oxidative stress, antioxidant defense enzyme and other mediators of cell damage/inflammation as suggested by the results of the present study.

## Conclusion

This study concludes that AuNPs cause the generation of oxidative stress and an impairment of the antioxidant enzyme glutathione peroxidase in rat brain. AuNPs cause the generation of 8-hydroxydeoxyguanosine (8OHdG), caspase-3, heat shock protein 70 (Hsp70), and IFN-γ, which may lead to inflammation and DNA damage/cell death. Gold nanoparticle treatment also caused a significant decrease in the levels of neurotransmitters such as dopamine and serotonin, indicating a possible change in the behavior of the treated animals (Figure 5).

## Abbreviations

AuNPs, gold nanoparticles; BBB, blood–brain barrier; GABA, gamma amino butyric acid; GPx, glutathione peroxidase; Hsp70, heat shock protein 70; 8OHdG, 8-hydroxydeoxyguanosine; IFN-γ, interferon-γ; MDA, malondialdehyde; NPs, nanoparticles.

## Competing interests

The authors declare that they have no competing interests.

## Authors’ contribution

All the authors have contributed equally. All the authors have read and approved the final manuscript.
